# Comment on Goudra et al. Anesthesia for Bronchoscopy—An Update. *J. Clin. Med.* 2024, *13*, 6471

**DOI:** 10.3390/jcm14217708

**Published:** 2025-10-30

**Authors:** Ana Belén Fernández, Manuel Gálvez, Rubén García

**Affiliations:** Anesthesiology and Postsurgical Intensive Care Department, Ntra Sra de Candelaria University Hospital, 38010 Santa Cruz de Tenerife, Spain; mgalpadilla@gmail.com (M.G.); rubengg.snv@gmail.com (R.G.)

We read with great interest the updated review by Basavana Goudra and colleagues [1]. This comprehensive update highlights the growing range of procedures within the field of interventional pulmonology. In particular, it focuses primarily on anesthesia management of three key techniques: the placement of endobronchial one-way valves in patients with severe COPD, bronchial thermoplasty for the treatment of severe asthma, and navigational bronchoscopy.

In recent times, the field of interventional pulmonology has grown significantly, along with the increasing need for an anesthesiologist for each of these procedures. This poses a real challenge for the anesthesiologist, as these patients often present with multiple comorbidities and have limited respiratory function. These include patients with severe bronchial asthma, advanced obstructive COPD, and emphysema—some requiring treatment with unidirectional endobronchial valves—and patients with interstitial lung diseases, lung neoplasms, or those who have undergone surgeries such as lobectomies. All of this represents a risk for the patient. Therefore, the anesthesiologist must be extremely cautious and maintain clear communication with the pulmonologist, who should provide details on the specific characteristics.

In our hospital, navigational bronchoscopy is not available, and bronchial thermoplasty for the treatment of severe bronchial asthma is not performed. However, endobronchial one-way valves are inserted through interventional techniques as a treatment for parenchymal volume reduction in cases of severe emphysema.

We have also encountered a few cases during the COVID-19 pandemic of spontaneous pneumothorax and persistent air leak, which were managed—sometimes as a last-resort measure—with endobronchial one-way valves. In these particular cases, the patients were in the intensive care unit, intubated, and on mechanical ventilation.

We have performed diagnostic and therapeutic bronchoscopies under deep sedation, including cryobiopsy sampling and the placement of a size 4 gel laryngeal mask, without complications [2,3].

We have also managed the anesthesia for the placement of endobronchial one-way valves using either general anesthesia or deep sedation, with size 4 or 5 laryngeal masks (i-gel). These procedures were also carried out without complications.

However, we were intrigued by your article, in which all patients underwent the procedure under general anesthesia with endotracheal intubation. Could you please share the reason for this choice?

Additionally, we were surprised by the frequent use of vasopressors in these patients. In our experience, the use of vasoactive amines has been almost unnecessary, as hemodynamic parameters have remained stable throughout.

It is also unclear to us whether it is the interventional pulmonologist who administers these anesthetic drugs. For example, you mention the use of remimazolam—could you clarify who is responsible for administering deep sedation for these types of procedures in your article and in your hospital?

We find the management of these patients in interventional pulmonology under deep sedation using a SuperNO_2_VA™ nasal mask quite interesting. This nasal mask has been shown to be superior to conventional oxygen support during deep sedation, both in colonoscopies and bronchoscopies [4,5]. However, your article does not mention its use—likely because most patients, as you state, undergo general anesthesia with endotracheal intubation.

Have you performed any procedures using this type of nasal mask?

Lastly, regarding sedation and the drugs used—specifically dexmedetomidine—we believe that there has been an increasingly uncontrolled use of this drug. In our practice, we use it for pulmonology procedures with a laryngeal mask or the SuperNO_2_VA™ nasal mask. We routinely administer a bolus dose over 20 min at 0.5–0.7 micrograms per kg intravenously. Alternatively, we use the intranasal route, administering 1–1.5 microg per kg with an atomizer followed by low doses of remifentanil and propofol TIVA infusions.

We do not understand the use of dexmedetomidine as the sole agent for sedation, as higher doses can cause adverse effects in these patients, who often have underlying cardiac conditions. Moreover, dexmedetomidine has virtually no analgesic effect, so the patient may react and experience an undesirable response to procedural stimuli.

Dexmedetomidine induces hypnosis in a manner similar to physiological sleep but has limited analgesic power at a dose of 0.5 micrograms/kg IV. It has been referred to as the “conscious sedation drug.” However, for procedures that require very deep sedation, dexmedetomidine alone is not a good option, as a painful stimulus may cause the patient to move uncontrollably, potentially leading to adverse events. Furthermore, high doses can also result in undesirable cardiovascular effects.

In our opinion, dexmedetomidine in interventional pulmonology should always be administered—provided it is not contraindicated by the patient’s clinical profile—in combination with another agent, such as remifentanil or propofol.
